# Umbilical Endometriosis Following Laparoscopic Cholecystectomy

**DOI:** 10.7759/cureus.14538

**Published:** 2021-04-17

**Authors:** Brittany Munkres, Melanie Espinet

**Affiliations:** 1 College of Osteopathic Medicine, New York Institute of Technology at Arkansas State University, Jonesboro, USA; 2 Family Medicine, Baptist Memorial Hospital, Memphis, USA

**Keywords:** cyclical bleeding, endometriosis, umbilical endometriosis, abdominal pain, abdominal bleeding, laparoscopic cholecystectomy, women's health, obstetrics and gynecology, umbilical mass, umbilical bleeding

## Abstract

Endometriosis is a painful disorder that most commonly causes dysmenorrhea and pelvic pain. It is defined as endometrial glands and stroma that occur outside of the uterus. Typically the lesions are located and contained within the pelvic cavity, however it can occur at multiple sites throughout the body. Here is a 28-year-old female who presents to an outpatient family medicine clinic with endometriosis of her umbilicus. CT scan revealed a 2-cm mass protruding continually through the umbilical stalk. The mass was then resected and pathology confirmed it to be consistent with endometriosis. Umbilical endometriosis is a rather rare finding, but still likely. This case presents differently due to the fact that the patient has never had a previous history of endometriosis or associated symptoms. The most likely suspected mechanism of the umbilical endometriosis would be the laparoscopic cholecystectomy done one year prior.

## Introduction

Endometriosis is a painful disorder where endometrial glands and stroma spread to places outside of the uterine cavity [1-3]. It typically is located in the pelvis, however, it can occur in multiple places such as the bowel, diaphragm and pleural cavity [2,4,5]. Symptoms can range from mild to debilitating. This ectopic tissue consists of fibrous tissue, blood, and cysts that can cause inflammation leading to symptoms such as dysmenorrhea, dyspareunia, chronic pain and even infertility in some cases [2,6]. Those considered high risk for endometriosis include nulliparity, early menarche, late menopause, shorter menstrual cycles, heavy menstrual bleeding, lower body mass index, and higher consumption of trans and unsaturated fats [7-9]. The pathogenesis of endometriosis is multifocal and there are various theories, however the most common theory is Sampson’s theory of retrograde menstruation [1,2,4,6]. This theory states that endometriosis occurs when endometrial cells flow backwards through the fallopian tubes and into the peritoneal cavity during menses [1,2,4]. Evidence supporting this theory demonstrates that the incidence of endometriosis is higher in girls with genital tract obstructions such as Mullerian anomalies that prevent proper drainage of menses through the vagina and increase reflux [2,8]. Although this may be the most popular theory there are many women with endometriosis who do not have an obstruction and vice versa.

## Case presentation

**Figure 1:**
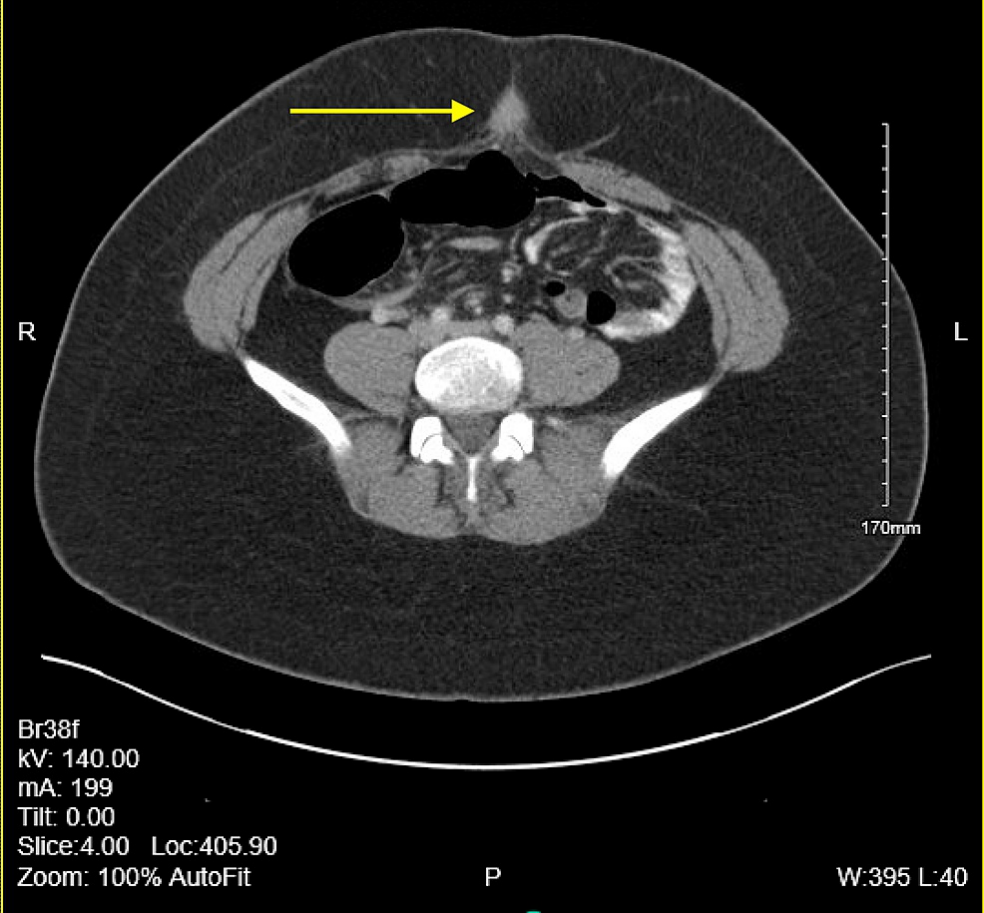
Computerized tomography (CT) scan 1

**Figure 2:**
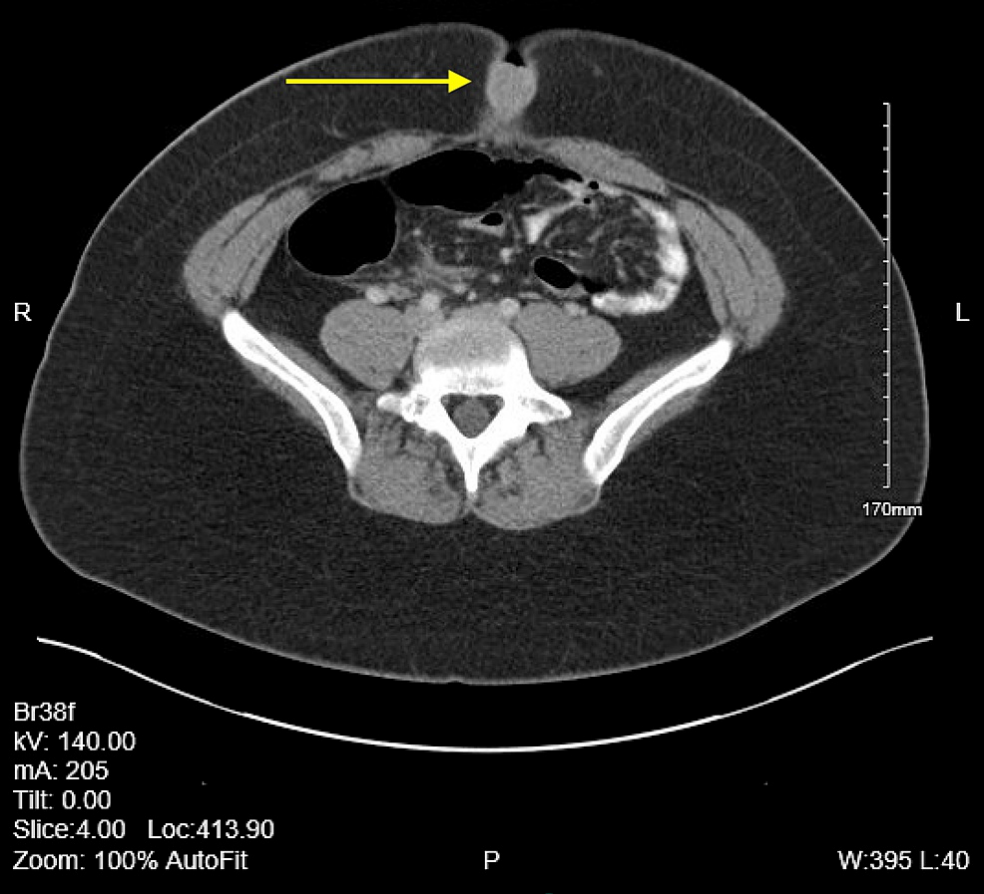
Computerized tomography (CT) scan 2

**Figure 3:**
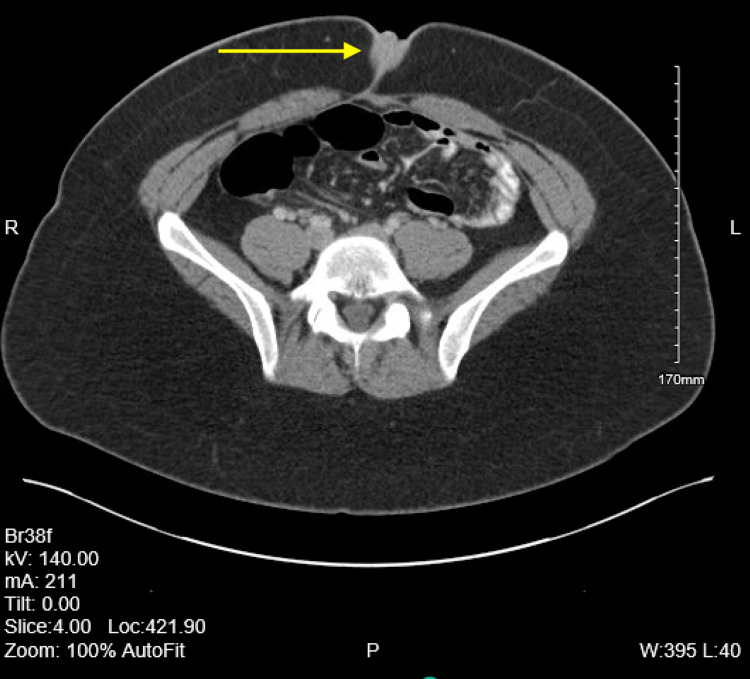
Computerized tomography (CT) scan 3

**Figure 4:**
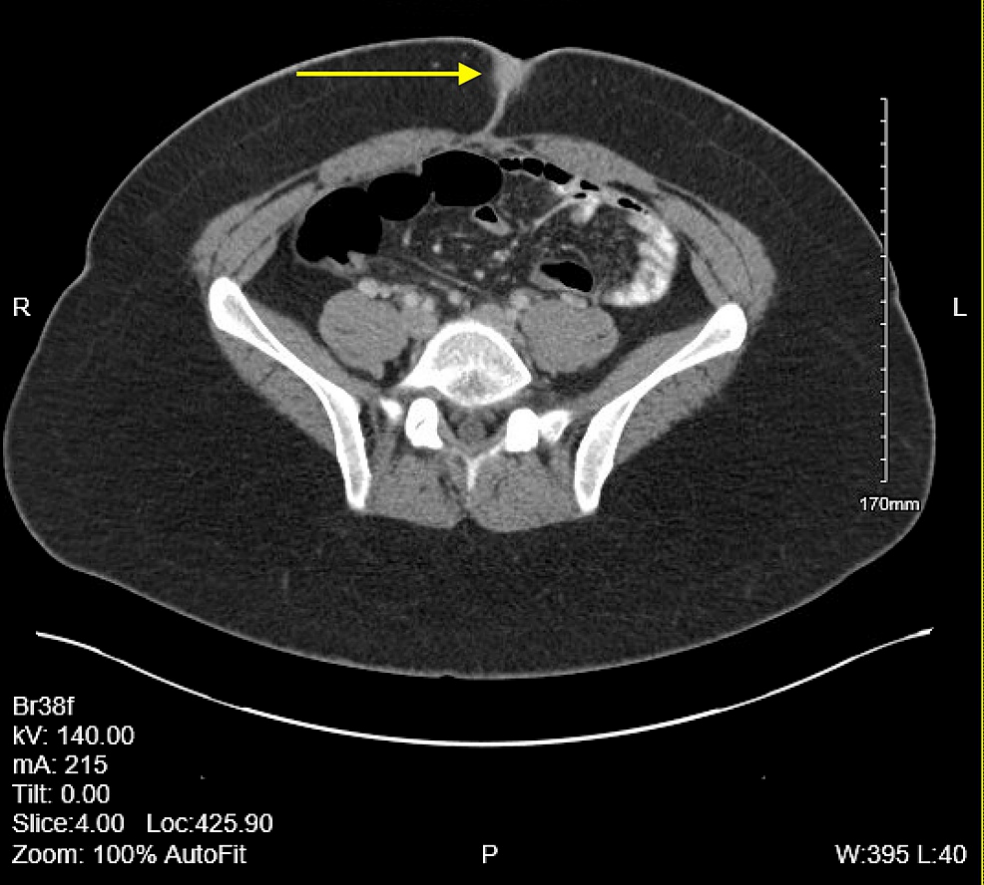
Computerized tomography (CT) scan 4

The patient is a 28-year-old female who presents to the outpatient family medicine clinic with multiple raised papular lesions protruding from her umbilicus that bleeds. She states that she noticed a mass at her umbilicus about a year ago right after her cholecystectomy that has continued to grow. Over the past year, the mass has become increasingly painful and bleeds only during her menstrual cycle. She denies having any other abdominal pain, appetite changes, changes in her bowel movement, urinary incontinence or retention, abnormal vaginal bleeding or discharge and fever. She also denied abdominal distension, anal bleeding, melena, hematochezia, nausea or vomiting. She has a past medical history of asthma, chronic back pain, Graves’ disease, and obesity. In addition she also has a past surgical history of an endoscopic retrograde cholangiopancreatography (ERCP) with sphincterotomy, through-the-scope (TTS) balloon with removal of sludge and a laparoscopic cholecystectomy performed one year prior. She denies tobacco, alcohol, or substance use and has no known drug allergies. On inspection of her abdomen there were scars from the previous cholecystectomy and multiple raised skin colored papular lesions on her umbilicus. The mass was easily visualized and there was no active bleeding from the mass or umbilicus during the exam. A computerized tomography (CT) scan with contrast of the abdomen and pelvis was performed. A 2-cm soft tissue mass involving the umbilicus can be seen in CT scans 1-4. CT scans 1-4 show the mass slowly protruding from the peritoneum to the surface of the abdomen (Figures 1-4).

The patient was then referred to general surgery where the surgeon found the mass to be consistent with endometriosis and that it warranted surgical resection at that time. The patient agreed to the surgery with complete excision of the umbilicus. The patient was prepped and draped in the supine position. A transverse ellipse was made in the periumbilical skin carried down with cautery through the subcutaneous tissue to the umbilical stalk, which contained the mass. The umbilical stalk at the fascial level was transected with cautery and removed from the field. Extra fascia was removed to take any extra margins away, due to some hard tissue still remaining. A 1.5-cm umbilical defect was left which was repaired with an interrupted horizontal mattress and running nonabsorbable sutures that came together under mild tension. The wound was then irrigated, dried and hemostasis confirmed. The patient was given an option to have reconstruction of her umbilicus done, however she declined. The patient recovered well postoperatively and continues to have no complaints.

## Discussion

Of all extrapelvic endometriosis, umbilical endometriosis comprises 0.5-1% [6]. Rarely this is due to laparoscopic surgery and the use of trocars [2], but in our patient her laparoscopic cholecystectomy played a part. Like the case report and systematic literature review done by Victory et al., they analyzed 122 patients with documented umbilical endometriomas, and only five of those patients had seeding due to laparoscopic or umbilical trocars. The presentation of our patient is consistent with that of endometriosis; cyclic pain, bleeding during menses, and abdominal pain. The definite treatment would be total excision of the tissue in the umbilical area and pathology report confirmed the presence of endometrial tissue [1,4,6]. There have been other treatments that have been proposed, such as pharmacological therapy, but most authors recommend surgical treatment as the treatment of choice [1,4-6]. If any endometrial tissue was left behind, the tissue would continue to grow over time. Therefore, clear margins would need to be excised during surgery to make sure no endometrial tissue is left [1,4,6].

Endometriosis can occur in patients who have no history of prior endometriosis [5], as was seen in this patient. Endometriosis should always be a differential diagnosis when dealing with females of a reproductive age who present with any of the symptoms; pelvic pain, abdominal pain, infertility issues, dysmenorrhea, or ovarian mass [3]. The peak age of endometriosis occurs in women 25-35 years of age. The definite diagnosis of endometriosis is a pathology report confirming endometrial tissue outside of the uterus [3]. This can be done via laparoscopic surgery taking biopsy samples and confirming the presence of the tissue [1]. That can be a rather extreme and invasive procedure. A majority of women who have endometriosis, complain of dysmenorrhea and infertility issues [2,5]. As a physician it is important to distinguish between primary dysmenorrhea and endometriosis as the cause. If a patient has recurrent infertility issues with no definite diagnosis, endometriosis should be high on the differential diagnosis.

## Conclusions

Although endometriosis is most commonly located in the pelvis it can occur virtually in any location throughout the body. In this case it was this patient’s umbilicus. There have been cases of endometriosis in lungs, diaphragm, skin, brain, bowel, and other sites. It is still unclear why it occurs in certain places in some women and not others. Due to these uncertainties physicians keep endometriosis as a differential when a patient presents with concerning symptoms anywhere in the body, not just in the pelvis. Although a small percentage, it is possible to show up in any place.

It is also important to note that possible complications of abdominal surgery could include future occurrence of endometriosis outside the pelvic region. While rare, this is something that could be anticipated when performing surgery on a woman who has been diagnosed with endometriosis. This patient had no known previous diagnosis of endometriosis so it would have been difficult to foresee her diagnosis. Even though umbilical endometriosis is a rare finding, this case demonstrates how abdominal surgery can cause seeding of endometriosis from the peritoneum to the umbilicus and cause a delayed cutaneous manifestation which demonstrates similar symptoms as pelvic endometriosis. The preferred treatment for umbilical endometriosis is surgical excision.
